# Extending the Graves’ Triad: Thyroid Eye Disease, Dermopathy, and Acropachy: A Clinical Response to Teprotumumab

**DOI:** 10.1210/jcemcr/luaf156

**Published:** 2025-08-13

**Authors:** Javier Lira, Paige Dixon, Mone Zaidi, Se-Min Kim

**Affiliations:** Department of Medicine, Icahn School of Medicine at Mount Sinai, New York, NY 10029, USA; Department of Medicine, Icahn School of Medicine at Mount Sinai, New York, NY 10029, USA; Center for Translational Medicine and Pharmacology, Department of Pharmacological Science, Icahn School of Medicine at Mount Sinai, New York, NY 10029, USA; Department of Medicine, Icahn School of Medicine at Mount Sinai, New York, NY 10029, USA; Center for Translational Medicine and Pharmacology, Department of Pharmacological Science, Icahn School of Medicine at Mount Sinai, New York, NY 10029, USA

**Keywords:** Graves’ disease, acropachy, dermopathy, ophthalmopathy, teprotumumab, insulin-like growth factor, IGF-1

## Abstract

Thyroid acropachy is a rare extrathyroidal manifestation of Graves’ disease. Due to a limited understanding of the pathophysiology, no specific treatment exists. Here we discuss such a case and its response to treatment.

A 25-year-old male with Graves’ disease, for which he had total thyroidectomy 2 years previously, presented with worsening double vision. He also reported swelling in legs, as well as pain and stiffness in his hands and feet. Physical examination revealed bilateral asymmetric proptosis and plaque-like lesions with nonpitting edema on the pretibial skin. Fusiform swelling of the fingers and clubbing of the fingernails and toenails were also noted. Laboratory findings showed elevated thyrotropin-receptor antibodies of 81.1 IU/L (0.0-1.75). He was euthyroid with levothyroxine supplementation. Bilateral hand X-rays noted soft tissue swelling and bilateral periostitis and subperiosteal bone formation. Teprotumumab was initiated for Graves’ ophthalmopathy, which improved his proptosis and orbital inflammation. Notably, the patient's pretibial myxedema, along with swelling in the bilateral metacarpals and phalanges, also improved.

This case is the first to demonstrate clinical improvement in thyroid acropachy following teprotumumab. This observation suggests that pathophysiology of thyroid acropachy may involve interplay between the TSH receptor and IGF-1 receptor signaling, similar to the ophthalmopathy.

## Introduction

Graves’ disease is an autoimmune condition caused by TSH receptor antibodies. About 20% to 40% of patients develop thyroid eye disease (TED) (Graves’ ophthalmopathy), presenting with exophthalmos, lid lag, strabismus, and lagophthalmos and progressing to optic neuropathy [1]. Among those with ophthalmopathy, approximately 4% to 13% also develop dermopathy, known as pretibial myxedema. This condition is marked by nonpitting edema, ranging from nodules, plaques, and polypoid lesions to elephantiasis-like edema in severe cases [2]. In rare instances, less than 20% of patients with Graves’ dermopathy may develop acropachy [3]. Acropachy is characterized by digital clubbing, soft tissue swelling of the hands and feet, and periosteal new bone formation [3]. Given its rarity, the pathogenesis of acropachy is poorly understood but can be inferred primarily by studying other extrathyroidal manifestations, especially ophthalmopathy.

The TSH receptor (TSHR) interacts with the IGF-1 receptor (IGF-1R) on orbital fibroblasts, leading to the production and accumulation of hyaluronic acid in the retroorbital space and the expansion of the extraocular muscles [1]. Tobacco use has also been associated with TED, possibly due to increased levels of proinflammatory cytokines [4]. In addition, the presence of the TSHR in the keratinocytes and fibroblasts of human skin supports the same concept behind the pathogenesis of Graves’ dermopathy [5]. In 2020, the Food and Drug Administration approved the use of teprotumumab, a monoclonal anti-IGF-1R antibody that blocks the interplay between TSHR and IGF-1R, for the treatment of TED following the demonstration of remarkable improvements in patients’ proptosis [6], and in theory this approach should be applicable to dermopathy and even acropachy.

While clinical improvement in Graves’ dermopathy has been reported in response to teprotumumab [7, 8], its effect on acropachy has remained unknown. Here, we report a patient who showed significant improvement in both dermopathy and acropachy in response to teprotumumab, suggesting that the interaction between TSHR and IGF-1R also plays a role in this rare extrathyroidal manifestation.

## Case Presentation

A 25-year-old male with a history of Graves’ disease, for which he underwent total thyroidectomy 2 years previously, presented with worsening blurry vision and watery eyes. He also reported nonpainful erythematous swelling on the anterior part of both of his legs and right thigh, along with puffiness, swelling, and stiffness in both hands and feet.

**Figure 1.**
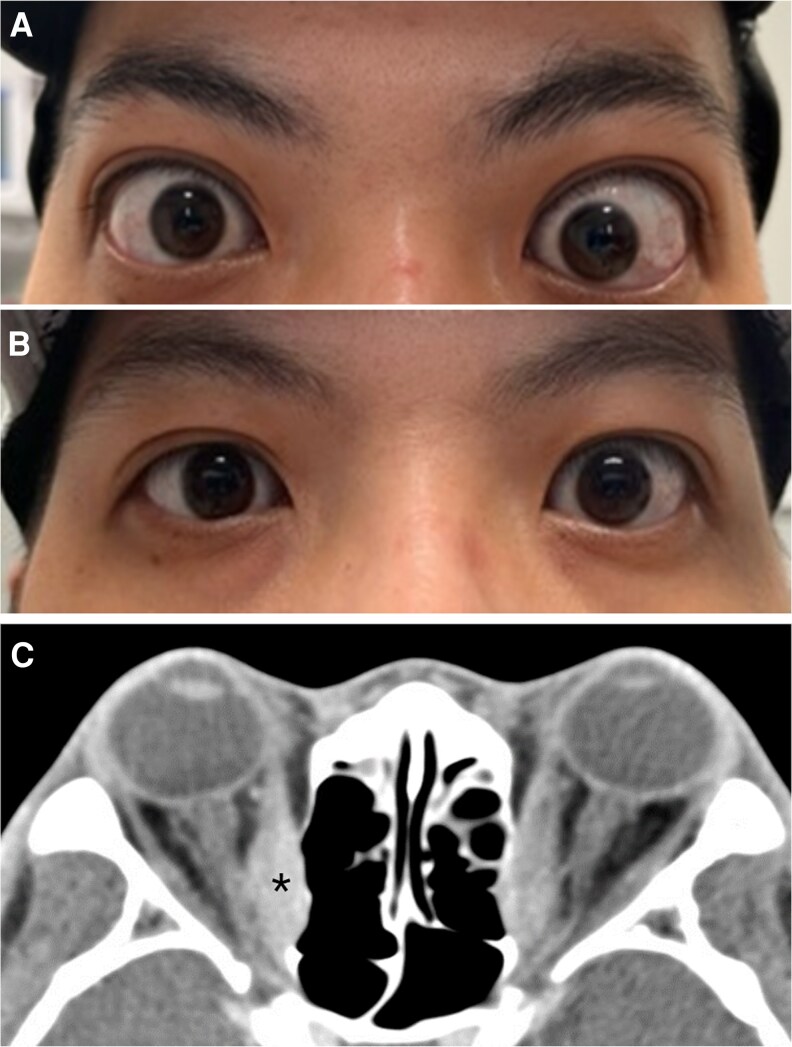
Graves’ ophthalmopathy with bilateral asymmetric proptosis with lid retraction (A) improved after teprotumumab (B). Computed tomography orbit showed bilateral proptosis, enlargement of rectus muscle (*), and crowding at the level of the orbital apex (C).

**Figure 2.**
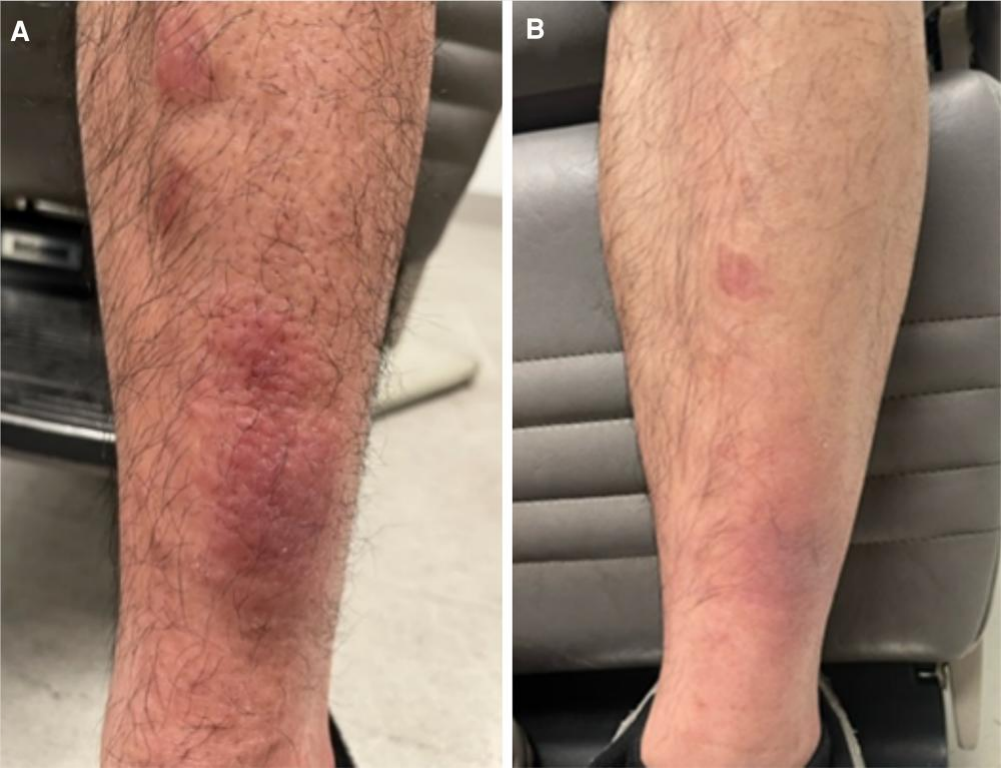
Graves’ dermopathy with nonpitting edema on lower extremity (A) showed improvement after teprotumumab infusion (B).

**Figure 3.**
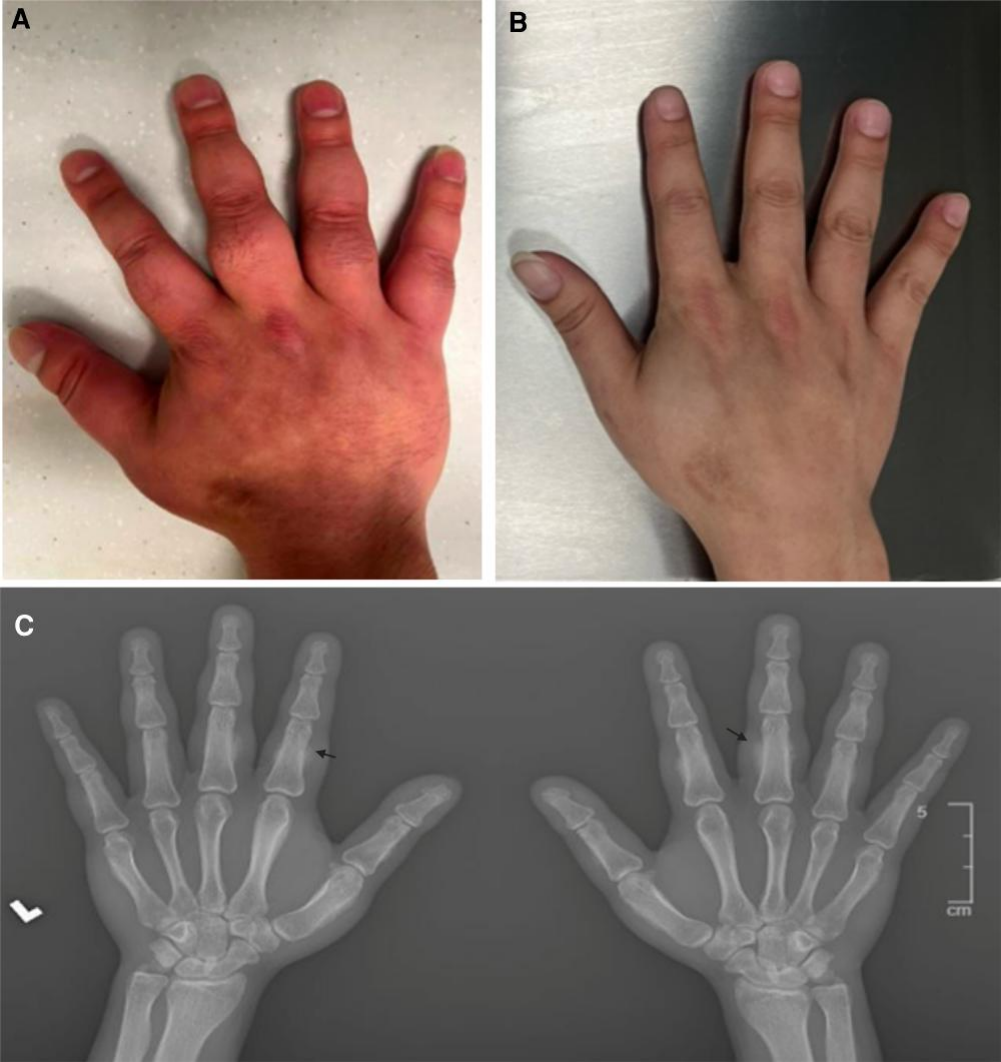
Graves’ acropachy with fusiform swelling (A) improved after teprotumumab (B). X-ray hands (C) showed soft tissue swelling and extensive bilateral periostitis and subperiosteal bone formation (arrows) in multiple metacarpals and phalanges.

On examination, there was bilateral proptosis (right 24 mm, left 25 mm with base 112), lid retraction, lagophthalmos, and mild periorbital edema with periorbital tenderness (Fig. 1). Erythematous, nontender, and raised plaques were noted on the bilateral anterior shins and right lateral thigh, with the largest plaque measuring 5 cm in diameter (Fig. 2). He had been seen by dermatologists at different institution and had a skin biopsy prior to the visit (see the pathology discussion later). Additionally, there was metacarpal involvement with proximal medial, distal phalangeal edema and associated nail clubbing bilaterally, and a limited range of flexion of the digits in both hands (Fig. 3).

He had been taking levothyroxine 150 mcg daily for postsurgical hypothyroidism and did not report any symptoms of hyperthyroidism or hypothyroidism. Upon further questioning, he admitted to smoking 4 cigarettes per day.

## Diagnostic Assessment

Thyroid function was normal with levothyroxine supplementation: TSH was 0.40 µIU/mL (0.40 mIU/L) (reference range: 0.4-4.20 µIU/mL; 0.4-4.20 mIU/L) and free T4 was 1.5 ng/dL (19.35 pmol/L) (0.8-1.5 ng/dL; 10.32-19.35 pmol/L). He had high levels of thyrotropin receptor antibody (TRAb) titer at 81.10 IU/L (0.00-1.75 IU/L). Computed tomography of the orbits revealed bilateral asymmetric orbital proptosis, with the left side greater than the right, and increased size of the medial rectus and bilateral inferior rectus muscles. There was crowding at the orbital apex with mass effect against the distal intraorbital course of the bilateral optic nerves, consistent with severe thyroid orbitopathy (Fig. 1). Bilateral hand X-rays demonstrated extensive bilateral periostitis involving multiple metacarpals and phalanges, consistent with thyroid acropachy (Fig. 3). The skin biopsy pathology report obtained from an outside institution confirmed an extensive, well-differentiated, myxomatous infiltration involving the dermis and subcutaneous fat, with collagen and abundant mucin (representing hyaluronic acid), consistent with pretibial myxedema.

## Treatment

The patient was counseled for smoking cessation. After a failed response to a short course of prednisone (50 mg daily for 7 days), teprotumumab therapy was initiated for his TED. A total of 8 infusions of teprotumumab were administered every 3 weeks, starting with an initial dose of 10 mg/kg over 90 minutes for the first infusion, followed by 20 mg/kg over 90 minutes for the remaining 7 infusions.

## Outcome and Follow-up

After the fifth infusion of teprotumumab, there was marked improvement in proptosis (right 15.5 mm, left 19 mm with base 103) and periorbital edema and resolution of conjunctival injection bilaterally (Fig. 1). There was a decrease in erythema and swelling of the pretibial myxedematous mass (Fig. 2). Additionally, the patient had a marked reduction in swelling of the bilateral metacarpals and phalanges and was able to flex the digits on both hands and grasp objects. Nail clubbing also improved (Fig. 3).

Repeat bilateral hand X-rays confirmed a marked reduction in adjacent soft tissue swelling and improvement in periostitis involving the first, second, and fifth metacarpals, as well as the first through fifth proximal phalanges, compared with the initial X-ray. TRAb titers decreased significantly to 33.4 (after the fifth infusion), 14.3 (at the completion), and then 8.01 (3 months after completion) IU/L (0.00-1.75 IU/L).

Overall, he tolerated teprotumumab infusions well except the temporary development of muffled hearing in both ears after the fifth infusion. The audiology assessment did not reveal any significant hearing impairment, and the muffled hearing spontaneously resolved.

## Discussion

The prevalence of thyroid acropachy in patients with Graves’ disease is estimated to be approximately 0.3% [9] and is almost always accompanied by other extrathyroidal manifestations, namely, TED and dermopathy. Patients with thyroid acropachy present with soft tissue swelling of the hands and feet, often accompanied by clubbing of fingers or toenails. Although it is usually painless, some patients may report stiffness or pain. Notably, joints or long bones are usually not involved, and the characteristic local warmth associated with increased blood flow in pulmonary osteoarthropathy is typically absent [9]. In the largest case series of 40 patients, 88% presented with clubbing, and 20% exhibited edema and thickening of the fingers and hands [3 ]. Additionally, approximately 50% of patients showed periosteal bone reaction on X-ray characterized by irregular, frothy, spiculated periosteal reactions in the mid-diaphyseal areas of the metacarpals and phalanges with soft tissue swelling [3, 10]. King et al [11] described findings from bone biopsies, revealing peculiar nodular fibrosis and subperiosteal bone formation with fibrosis of the intervening marrow spaces.

The pathophysiology of thyroid acropachy remains poorly understood. However, there is a potential similarity with other extrathyroidal manifestations, involving autoimmune-mediated dysregulation of periosteal fibroblast and mucopolysaccharide synthesis and deposition. High titers of TSHR antibodies have been consistently identified in all affected patients [3]. Another aspect under consideration is the potential contribution of bone cells in the inner osteogenic periosteal layer. Subperiosteal bone formation in thyroid acropachy can be triggered by local inflammation or fibroblast differentiation into osteogenic cells. In addition, preclinical evidence of direct skeletal effects of TSH suggests a potential direct role of bone cells in acropachy. Previous studies have documented TSHR expression in human skeletal tissue and bone cells [12, 13], and TSH was shown to exert inhibitory action in osteoclastogenesis and bone resorption [14]. Furthermore, some studies suggest that TSH may promote osteoblast-mediated bone formation directly through the activation of the Wnt pathway [15] or by activating the IGF-1 pathway via β-arrestin [16 , 17].

Given the overlap in the pathophysiology of Graves’ acropachy and Graves’ ophthalmopathy, it is plausible that tobacco smoking, the most important risk factor for the development of Graves’ ophthalmopathy [18], may also play a role in the development of Graves’ acropachy. There are several established mechanisms through which smoking contributes to the development of Graves’ ophthalmopathy. First, smoking is associated with increased titers of TRAb, which acts on TSHR of orbital fibroblasts, leading to upregulation of hyaularonic acid synthesis and adipogenesis, thus increasing tissue expansion and remodeling within the orbit [19]. During treatment with antithyroid drugs, smoking status was correlated with the extent of TRAb reduction, while the number of cigarettes smoked was negatively associated with the change of TRAb levels [20, 21]. Independently of TRAb-mediated effects, smoking is also associated with upregulation of immediate early genes and proinflammatory cytokines such as IL-1β and IL-6 in orbital fibroblasts, further promoting inflammation and adipogenesis [22]. Additionally, cigarette smoke extract has been shown to induce oxidative stress in orbital fibroblasts, leading to increased expression of fibrosis-related genes such as connective tissue growth factor, TGF-β1, and IL-1β [18]. Finally, smoking causes tissue hypoxia and activation of hypoxia-inducible factor-1 pathways in orbital fibroblasts, which enhances adipogenesis as well as angiogenesis via production of vascular endothelial growth factor secretion [23], altogether possibly contributing to the development of Graves’ acropachy. The decrease in TRAb titer in this case is most likely attributed to smoking cessation rather than the treatment effect of teprotumumab.

Due to the uncommon occurrence of acropachy, its benign clinical course, and the limited understanding of its pathophysiology, there is currently no specific treatment for acropachy. In cases where painful periostitis arises, management may involve pain relief with anti-inflammatory agents. This case is unique as it is the first example of clinical improvement in patients with thyroid acropachy following teprotumumab treatment. This observation also indirectly implies that the pathophysiology of thyroid acropachy may encompass the interplay between TSHR and IGF-1R signaling, akin to Graves’ ophthalmopathy. In addition, it also highlights the potential direct impact of TSH signaling on bone cells, as suggested by preclinical findings [24].

## Learning Points

- Thyroid acropachy is a rare extrathyroidal manifestation of Graves’ disease that may cause painful symptoms and physical impairment.
- This patient developed TED, dermopathy, and acropachy after total thyroidectomy, highlighting that Graves’ disease is a systemic condition.
- Smoking cessation should be advised for patients with Graves’ disease, as it can trigger all extrathyroidal manifestations.
- The marked improvement with teprotumumab, which targets the IGF-1R, suggests that the IGF-1R plays an important role in both dermopathy and acropachy.

## Data Availability

Data sharing is not applicable to this article as no datasets were generated or analyzed during the current study.
